# The decision-making process leading to deep brain stimulation in men and women with parkinson’s disease – an interview study

**DOI:** 10.1186/1471-2377-14-89

**Published:** 2014-04-25

**Authors:** Katarina Hamberg, Gun-Marie Hariz

**Affiliations:** 1 Department of Public Health and Clinical Medicine, Family Medicine, Umeå University, Umeå 90187, Sweden; 2 Department of Community Medicine and Rehabilitation, Section of Occupational Therapy, Umeå University, Umeå 90187, Sweden; 3 Department of Pharmacology and Clinical Neuroscience, Section of Neurology, Umeå University, Umeå 901 87, Sweden

**Keywords:** Parkinson’s disease, Deep brain stimulation, Patients’ perspective, Gender, Equity in health, Interviews

## Abstract

**Background:**

Deep brain stimulation (DBS) is an established treatment for patients with advanced parkinson’s disease (PD). Research shows that women are under-represented among patients undergoing DBS surgery. This may be due to gender-biased selection of patients, but patients’ wishes and attitudes may also contribute. This study investigated the decision making process to undergo DBS from the patient’s perspective, and explored any gender patterns in the participants’ decision-making.

**Methods:**

All patients operated on with DBS for PD at the University Hospital of Northern Sweden between January 2002 and April 2010 were invited to an interview study. In this way 39 patients were recruited, 31 men and eight women. Three additional women, operated elsewhere, were recruited to acheive a more gender-balanced sample. In a mixed-method analysis, the interviews were analysed according to the constant comparison technique in grounded theory and descriptive statistics was used to present demographics and compare categories.

**Results:**

Three different approaches to DBS were identified among the patients. ‘Taking own initiative’, included 48% of the patients and implied that the patients’ own initiatives and arguments had been crucial for having surgery. ‘Agreeing when offered’, and accepting DBS when suggested by doctors embraced 43%. The third approach, ‘Hesitating and waiting’ included < 10% of the patients. Most of the men were either ‘taking own initiative’ or ‘agreeing when offered’. The 11 women were evenly distributed in all three approaches. Among the interviewed, more women than men expressed strong fear of complications and more women consulted friends and relatives prior to deciding about DBS. Half of the patients had held a leadership position at work or in another organisation, and among patients ‘taking own initiative’ the proportion with leadership experiences was 80%. At time for surgery ten men but no woman were professionally active.

**Conclusion:**

This study suggests that many patients with advanced PD have to argue and struggle with their clinicians in order to be referred to a DBS-team. The study further suggests that patients’ wishes, behaviour and position in society may all contribute to the skewed gender distribution among patients treated with DBS.

## Background

Parkinson’s disease (PD) is a progressive neurodegenerative disorder affecting both men and women. It is characterized by motor symptoms as well as cognitive, behavioural, autonomic and other non-motor symptom [1]. There is no cure for the disease and the medical and increasingly used surgical treatments aim at alleviating the symptoms. Over the last decade, deep brain stimulation (DBS) of different brain targets, mainly DBS of the subthalamic nucleus (STN), has become an established surgical procedure for patients with advanced PD [2,3]. According to estimates, to date more than 100 000 patients worldwide have had DBS [4].

Research reviews on the incidence of PD suggest that in Western populations the male to female incidence ratio is 3/2 [5,6]. There is, however, a significant heterogeneity between studies. While many investigations show that PD affects more men [7-10], others report an equal gender distribution [11,12]. In a recent population-based study from northern Sweden no significant gender difference was found in the incidence of PD [13].

Several reports indicate that women with PD are under-represented among those referred for surgery [14-16]. A recent review on the gender distribution among PD patients undergoing the most common neurosurgical procedure of the last decade, i.e., STN DBS, showed that only about one third were women [17]. Hence, the proportion of male patients who are treated with DBS seems to exceed commonly reported male to female ratios of patients with PD.

It has been discussed whether gender-biased decisions and selection of patients are the reasons behind the low percentage of women with DBS [6,14-18]. Other suggestions have been that patients’ own wishes and attitudes may lead to gender differences in the use of DBS. For example, it has been put forward that women might be more “afraid” of surgery and as a result abstain from operation if offered [6,17,19].

Because DBS is an elective, symptomatic and non-curative surgery, the final decision to undergo DBS has to be taken by the patient. To the best of our knowledge, there is a lack of research about the patients’ ponderings and thoughts before making their decision. In this study we interviewed PD patients who had undergone DBS about their experiences, wishes and considerations that led to their decision to undergo surgery. The aim was to investigate the decision-making process from the patient’s perspective and to explore any gender patterns in the participants’ decision-making.

## Methods

In Sweden, patients with symptoms suggesting PD are supposed to be referred to a neurology clinic for diagnosis, treatment and follow-up. Elderly patients with PD are cared for by geriatricians. If treatment with DBS seems to be an alternative, a referral for assessment is sent to a multi-disciplinary DBS-team at the neurosurgery department in the catchment area. There are no private hospitals in Sweden that carry out neurosurgery for PD.

### Participants

Patients operated on with DBS for PD at the University Hospital of Northern Sweden between January 2002 and April 2010 were invited to participate in this interview study. In total 50 consecutive patients were operated during this period, 38 men and 12 women. Two patients (1 woman) had died of causes unrelated to surgery, and a letter of invitation was thus sent to 48 patients. After a reminder, 31 men and eight women agreed to participate. To include some additional women, we asked representatives from the Parkinson’s Disease Society, if they knew of other women treated with DBS at other hospitals who could be asked. This provided three additional women. In total, 42 patients were interviewed, 31 men and 11 women, all operated on between January 2004 and April 2010. The local ethical board at Umeå University approved the study (D.no: 2010-97-31 M).

### Data collection

Data were collected through qualitative interviews conducted by either author. Most interviews were performed face-to-face in the patients’ home or at the clinic. Five patients living in remote areas were interviewed by telephone.

The interviews were thematically structured with open-ended questions concerning broad areas in relation to PD and its treatment. The main areas were the course of the disease before surgery, considerations about surgery, the operation itself, and symptoms and life with PD after the surgery. In this article we focus on the patients’ experiences of the initiation and decision processes that led them to undergo DBS surgery. Interview questions related to this focus included: “How did you get to know about DBS?”, “Who suggested DBS as a treatment for you?” and “What considerations were most important when you decided to undergo DBS?” The interviewer tried to facilitate the narrative by follow-up questions, such as “Please could you give an example?” and “What happened then?” Each interview lasted 60 to 140 minutes, was digitally recorded and transcribed verbatim.

Each patient also filled in a short questionnaire about socio-demographic information.

### Mixed method analysis

The data was analysed using a mixed method approach [20]. A qualitative analysis of the interviews was combined with the use of descriptive statistics to present demographics and compare categories.

#### Qualitative analysis

According to qualitative research design [21], preliminary analyses of the transcriptions were conducted in parallel with the interview process. In this way the authors could successively refine the interview questions, learn and reflect during the interview process and be alert when new aspects were described.

The interviews were analysed according to the constant comparison technique in grounded theory [22]. The analysis contained the following steps:

1. The researchers separately read and coded three interviews and then met to compare codes and outline preliminary categories that included the content and meaning of the patients’ experiences. Another three interviews were then coded and compared, and this process of sorting the data continued until all interviews were analysed.

2. Each interview was re-read and condensed into a case narrative of two to three pages of text reflecting the previous coding and the essentials of the patient’s story.

3. The case narratives of all 42 patients were systematically compared for similarities and differences regarding the path to surgery, i.e., circumstances and thoughts that were part of the decision about undergoing DBS were categorised and compared between all narratives. This comparison resulted in the following three categories, i.e., three different approaches in the decision process towards DBS: *‘Taking own initiative’* for DBS, *‘Agreeing when offered’*, that is, accepting neurosurgery when suggested by the physician, and *‘Hesitating and waiting’* before accepting to undergo surgery. Eleven dimensions with importance for the participants’ approaches were also delineated.

4. In the final step, the whole interviews were re-read to ensure that the categories and interpretations could be re-contextualized into the interviews, that is, that the results were grounded in the data.

The categories and dimensions are described in text and illustrated with the use of quotations from the participants.

#### Statistical analysis

The distribution of participants in the three categories is compared by calculation of proportions. Demographic data are presented as numbers, mean ± SD, and range. Comparisons of group mean values were performed by one-way analysis of variance (ANOVA), followed by the Bonferroni *post-hoc* analysis. When variance was not equal in the Levine’s test, Welch one way ANOVA followed by the Games Howell *post-hoc* test was used. A p-value <0.05 was considered statistically significant. Statistical analyses were performed using the SPSS software version 17.0 (SPSS, Inc, Chicago, IL).

## Results

### Demographics

The demographics of the 42 patients (74% men) are presented in Table 1. Age at surgery ranged from 41 to 79 years, and the women were significantly younger than the men when they underwent surgery (p = 0.046, not shown in table). The majority of participants were living with a spouse. Most participants had high school or university education, but nine men had no formal education after primary school. At the time of surgery 10 patients (all men) were still able to work half- or full-time, 15 were full-time sick-listed (6 men and 9 women) and 16 had passed retirement age (14 men and 2 women). Eighteen men (58% of the men) and four women (36% of the women) had, or had had, a leadership position at work, in a trade union or in a non-governmental organisation (NGO), and 17 men (55% of men) and 10 women (91% of women) were members of a PD society.

**Table 1:** Gender, age, civil status, level of education, employment status at the time of surgery, having had a leadership position, membership in a PD-society, according to the three categories ‘Taking own initiative’, ‘Agreeing when offered’ and ‘Hesitating and waiting’

	**Whole group**	**‘Taking own initiative’**	**‘Agreeing when offered’**	**‘Hesitating and waiting’**
	**N = 42**	**N = 20**	**N = 18**	**N = 4**
		**Mr 1–16, Ms 17-20**	**Mr 20–34, Ms 35-38**	**Mr 39, Ms 40-42**
**Gender:**				
Men: N (%)	31 (74)	16 (80)	14 (78)	1 (25)
Women: N (%)	11 (26)	4 (20)	4 (22)	3 (75)
**Age: †**	*mean ± SD(range)*	*mean ± SD(range)*	*mean ± SD(range)*	*mean ± SD(range)*
Age at diagnosis (y)	52.6 ± 10.2(33–70)	55.7 ± 10.3(33–70)	50.4 ± 9.6(35–68)	47.8 ± 10.7(38–63)
Age at DBS surgery (y)	61.3 ± 8.4(41–79)	64.6 ± 7.4(49–79)	59.3 ± 7.3(49–71)	54.3 ± 12.4(41–71)
Age at interview (y)	64.1 ± 8.2(44–81)	67.0 ± 7.1(50–81)	62.3 ± 7.6(50–73)	57.5 ± 12.0(44–73)
Time between surgery and interview (y)	2.8 ± 1.9(0.5-8)	2.4 ± 1.5(0.5-5)	3.1 ± 2.3(0.5-8.0)	3.25 ± 1.9(2–6)
**Civil status:**				
Cohabitant N (%)	29 (69)	15 (75)	12 (67)	2 (50)
Single N (%)	13 (31)	5 (25)	6 (33)	2 (50)
**Level of education:**				
Primary school N (%)	9 (21)	4 (20)	5 (28)	0
High school N (%)	17 (41)	7 (35)	6 (33)	4 (100)
University N (%)	16 (38)	9 (45)	7 (39)	0
**Employment status at time of operation:**				
50- 100% Work N (men/women)	10	4/0	6/0	0/0
100% Sick-listed N (men/women)	15	2/3	4/3	0/3
100% Retired N (men/women)	16	10/1	3/1	1/0
Data missing N	1	0/0	1/0	0/0
**Leadership position** N (%)	22 (52)	16 (80)	6 (33)	0
**Member of a PD society** N (%)	28 (67)	14 (70)	11 (61)	3 (75)

The fictitious names of patients in each category are shown in table-head.

**†** = No significant differences were found between patients in the three categories, with respect to ‘Age at diagnosis’, Age at DBS surgery’, Age at interview’ and ‘Time between surgery and interview’.

### Interviews

At the time of the interview the participants appeared to have no difficulties in recalling and they were detailed in their narratives. They reported how they had received and perceived information about DBS, and they described their own reactions, questions and concerns regarding neurosurgery.

As shown in Table 1, all but one of the 31 men were represented in the categories ‘Taking own initiative’ and ‘Agreeing when offered’ while the 11 women were evenly distributed in all three categories, including in ‘Hesitating and waiting’.

Eleven dimensions, i.e., circumstances with importance for the patients’ approaches towards DBS, were identified. The patients’ own ‘*level of knowledge about DBS’* and ‘*the doctor’s attitudes’*, were crucial dimensions that framed most narratives and coloured the patient’s ponderings*.* Other important dimensions concerned *‘severity of the disease’*, ‘*operation risks’*, *‘managing own worries’*, and ‘*support from friends and relatives’.* For some participants, ‘*inspiration from other patients’,* ‘*significant contacts’* with people with influence on patient care, *‘age’*, ‘*own technical skills’* and the ‘*exclusiveness of DBS-treatment*’ had influenced their approach towards DBS.

In the following, the categories and dimensions are presented and illustrated with quotations from the participants. The participants are given fictitious names from Mr One to Ms Forty-two.

### Taking own initiative

Twenty participants (4 women) belonged to this category implying that their own initiative, effort and arguments had been important for having surgery. Nine of them (45%) had university degree and 16 (80%) were, or had previously been, in a leading position at work, in a trade union or a NGO (Table 1). A majority were members of a PD-society. Four men (25% of the men) were still working, at least half-time, at the time of the operation.

Two subcategories emerged, ‘demanding and arguing’ and ‘simply asking’. In both the subcategories the majority of patients were well informed about DBS through the media, the Internet, the PD-society, or friends, and their *level of knowledge* was high. The intensity in their initiatives and measures varied though, and was related to *the doctor’s attitudes* and his or her responses to their questions or suggestions*.*

#### ‘Demanding and arguing’

For 12 patients (9 men, 3 women) there had been great difficulties with numerous ‘demanding and arguing’, to convince their physicians about DBS. These patients had been forced to increase their knowledge about PD, use their creativity, and involve friends and relatives, and in one case even threatening to report the clinic to the healthcare authorities, in order to be granted a referral to a DBS team. All dimensions occurred in this subcategory.

Mr One is an example: ten years after being diagnosed with PD the *severity of the disease* was hard to manage with medication and he suffered greatly from “off” time, tremor and spasms, “I had to stop the car 85 times on a distance of 30 kilometres.*”* He had a high *level of knowledge about DBS* and was convinced that neurosurgery would be a good option for him. However, despite repeated requests he was not referred for assessment for surgery until he lost his patience and left his ordinary clinic:

“… I was fed up after three years of asking when once again the neurologist answered my question about DBS by saying that there were still many drugs to test… I told him I had enough of his pills… I managed to get an appointment at another hospital where it was decided about surgery.”

Ms Eighteen had a similar story. She suffered severe side-effects from her medication and needed assistance both day and night “I was either a propeller or totally stiff…” She asked for surgery but did not receive a referral. She tried herself at another clinic but was refused an appointment: “My life nearly broke down”. A friend of hers, active in the same PD society, helped her and wrote a letter to a third clinic where she was accepted for surgery. Ms Eighteen was *knowledgeable* and determined but her experiences illustrate that *support from friends* was sometimes crucial on the way to DBS treatment.

So was *support from relatives*. Some relatives had pushed for changed treatment and surgery when the patients were too tired or felt bad at persistently asking. Ms Nineteen said that there would have been no operation had it not been for her daughter’s demands: “She insisted on a referral and stood behind the doctor to control what he wrote.”

*Significant contacts,* i.e., contacts with people with influence in healthcare, had been vital for a few participants in the attempts to get through to DBS. Ms Seventeen had frankly asked a neurologist, who was speaking at a meeting of the PD-society, to send a referral for DBS assessment, which he also did. Her ordinary doctor had not, although she had raised the issue several times with him. Another example was a male patient who contacted an old schoolmate, nowadays a PD-specialist, to get advice on how to argue for surgery.

*Own technical skills* or interests made surgery with advanced technology understandable or appealing for a few of the men. Mr Four was engineer and heard about DBS on TV. He became interested and determined: “It was very interesting because I was familiar with what they talked about. Frequency, amplitude, voltage, current… It is easier to make a decision when you understand.” Having had own experiences with practical skills such as drilling could also make potential risks comprehensible: “I have done a lot of drilling so I know how easy you can just go too far…” (Mr Two).

*Age* had been an obstacle for some older participants. At the age of 71, Mr Twelve thought that he was too old because “They had decided on an age limit at 70 years”. He felt that he had no time to wait and used all knowledge he had gained on the Internet and in the PD-society to convince the doctors that in his case the pros with DBS were really stronger than the cons.

A few patients who pushed intensively for DBS were *less knowledgeable* and had little insight into the procedures and risks associated with the treatment. Even so they were *inspired by other patients,* who had been operated on with good results. Mr Two said: “For some months I pushed heavily… I had seen how the men were sitting and eating without problems while I was sitting there shaking.” Like two other men in this category, he was also tempted by the *exclusiveness* he saw in this treatment. It could not be offered to many – it was too advanced and expensive: “… something that not all have the opportunity to experience.”

Even if they were arguing for DBS, most patients simultaneously described worries and had considered *operation risks*, including complications and negative side effects. In one way or another, they *managed their worries* by putting their trust in the skills of the doctors, “I relied entirely on the surgeon” (Ms Nineteen). They also tried to balance between their worries about surgical complications and their great sufferings, by focussing on the chance for some improvement and for regaining own control: “The thoughts of operation might be frightening, but that changes if it means a chance to do something about one’s own situation… “(Mr One).

#### ‘Simply asking’

For eight patients (1 woman) it had been sufficient enough to simply ask their clinician about surgery to start a dialogue about DBS, and to be referred for assessment at a neurosurgery department. These patients described their *doctors’ attitudes* as attentive and respectful. When being informed and assessed as suitable candidates for surgery, all eight decided that they wanted DBS and four went through with surgery at once. The four others did not themselves consider the *‘severity of the disease’* as advanced enough to balance the *‘operation risks’* and they were promised, and also received, a new assessment later. They appreciated the relation and communication with their doctors, and felt very involved and in control of the decision process and their own treatment.

### Agreeing when offered

Eighteen participants (4 women) belonged to this category (Table 1). They agreed to neurosurgery when the physician offered it but had not themselves asked about DBS. Seven had a university exam (39%), six were or had been in a leading position at work or elsewhere, and 11 were members of a PD-society. Six men (43% of the men) were working part- or full-time at the time of surgery.

For the majority who took this approach to the decision-making, the *severity of the disease* implied that the suggestion for DBS came as a great relief. They described that they had come to “the end of the road” (Ms Thirty-seven) and would have accepted any treatment with a chance for improvement. “I had home-help six times a day to manage to eat, wash myself, dress…” (Mr Twenty-one).

The *level of knowledge about DBS* varied. Many patients had heard about DBS and some had been hoping for surgery, but none had shared their thoughts with their doctor. Still, when the doctor suggested DBS they were prepared and it was rather easy to accept:

“I had seen DBS-operations on TV and I read an article that I cut out and saved… But a long time passed and it was not until the neurologist asked me that it became real…” (Mr Thirty-four).

Others had minor knowledge about DBS or did not even know that such a treatment existed. When offered and informed about DBS, they needed time to think, weighting opportunities and *operation risks*. Mr Twenty-five, a well-educated technician, said: “I did not know what DBS was, so I had to find out first. Then I had problems deciding what to do… It was a difficult decision…”

To *manage their worries* about *operation risks,* most patients ‘agreeing when offered’ reacted like the patients in the previous category. They calculated the risks with the chance for improvement and they put their trust in the surgeon’s skills. In addition, some tried to keep the hazards at distance “I tried not to think that much about negative consequences” (Mr Twenty-seven), or avoided information that might cause worries “I did not go out on the Internet until after the operation” (Mr Thirty-one). For others the *severity of the disease* was horrendous and fear for treatment risks faded away. Ms Thirty-five exemplified this: ”Before… When people talked about their DBS-operation I had to leave the room in order not to faint…” Later, when she was offered DBS her situation was poor and she reacted totally different: “Everything was terrible with side-effects and spasms. The only thing I wanted was to have the operation done fast…”

Mr Twenty-three was an outlier since in his case the doctor initiated the surgery although the patient himself thought of his symptoms as pretty mild and he managed to work full-time. He was *inspired by other patients* though, who were operated on with good results, and he felt that he “should take the chance.”

### Hesitating and waiting

This was the smallest category including one man and three women. All had passed high school, none had had a leading position, and three were members in a PD-society (Table 1).

When recommended for DBS by their clinicians these patients reacted with apprehension and hesitation. They declined to be referred to a DBS team, at least in the near future. They expressed strong *worries* and fears of brain damage and it was a long time before they saw themselves as ill enough to accept the *operation’s risks*. In contrast to most other patients interviewed, they were not able to *manage their own worries* by putting trust in the surgeon’s skills. Two of the women had high *level of knowledge*, were well informed about DBS, and they also personally knew patients who were successfully treated with DBS. Still, their ponderings were all about the dangers with DBS and not about possibilities for improvement.

The narrative by Ms Forty-one was illustrative. After a few years with PD the side effects of her medications became severe and her neurologist suggested DBS several times. She refused operation and described strong fear: “The thought of operation scared me to hell… I was afraid of not being able to lie on the operation table… I thought, what will happen if I get those spasms when they dig into my brain?”

When Ms Forty-one finally accepted operation she had severe hyperkinetic movements most of the day and had lost weight. The operation was successful, and at the interview, she reflected on why she did not accept DBS earlier on:

“I was not aware of how bad I was… I have seen a video-film where I’m thin and skinny. I cannot sit on a chair because of all the movements and instead I slide under the table. The sweat runs… Seeing this film is hard for me… I was totally occupied by carrying on… I was in a glass bubble, kind of… ”

Also, the two other women in this category described that they had successively become used to severe symptoms: “I could only move normally for about one hour a day. I held myself down on the kitchen bench not to fall on the floor when I had dyskinetic movements. But stiffness was the worst, I was a prisoner in my own body” (Ms Forty-two).

While analysing these stories it seems as if some patients with difficult symptoms had lost all ability to plan and make decisions, they just struggled to survive from hour to hour.

Mr Thirty-nine, the only man ‘hesitating and waiting’, was reluctant to go through surgery when he was first asked. He was retired but worked daily on his house and thought that he was too healthy to take the risks. However, when he was repeatedly recommended for DBS, and his family took a persuasive standpoint, he decided to go ahead.

## Discussion

This study investigated the decision-making process in view of going through DBS for PD from the patients’ perspective, and explored whether there were any gendered patterns in this process. Three different approaches to DBS were identified: ‘Taking own initiative’, with the subcategories ‘demanding and arguing’ and ‘simply asking’, was the most common approach, and accounted for 48% of the patients; ‘Agreeing when offered’ and accepting DBS when proposed by the clinician was described by 43% of the patients; and ‘Hesitating and waiting’ included about 10% of the patients. The patients’ approaches were framed by their own knowledge about DBS, their doctor’s attitude to this treatment, the severity of their disease, and how they managed their own worries about complications. Support from relatives, friends, and significant people with influence in healthcare were important for some patients, as was inspiration from other patients that had been operated on with good results. Both sexes were represented in all three approaches, but while 30 of the 31 men were either ‘taking own initiative’ or ‘agreeing when offered’, the 11 women were evenly distributed in all approaches, including in ‘hesitating and waiting’. At the time of surgery, ten of the men were working half- or full-time compared to none of the women. In addition to gender, other social determinants seemed to be important for the patients’ approach and admission to DBS. Half of the operated patients had, or had had, a leading position at work, in a trade union or a NGO, and among patients ‘taking own initiative’, the proportion of those with leadership experiences was 80%.

Inasmuch as DBS is nowadays established as an efficient treatment in selected patients with advanced PD [3], it was surprising that so many (12 out of 42) of our participants had to break through resistance from their neurologists and geriatricians before being referred for assessment. One might argue that even if the patients demanded DBS, this treatment might not be suitable for them from a medical point of view. However, since the patients were seen as candidates for DBS when assessed by the DBS team, such an interpretation seems less reasonable. Part of the explanation could be that some patients were operated on in 2004 when DBS was probably not as well established as today. Still, some patients who had been forced to insist repetitively to obtain a referral were operated as late as 2010, indicating that there are still contradicting views about DBS among clinicians. This may create confusion among patients and may lead to unequal care.

In line with recent research about DBS in the treatment of dystonia [23], the patients in our study, who had tried for a long time to persuade their doctors to refer them for assessment for DBS, described despair, being neglected and dismissed. They succeeded by way of unusual and sometimes extreme measures, such as by threatening to complain to health authorities if the clinician did not send a referral, or by convincing PD-experts whom they met serendipitously at lectures to send a referral. A question that is not possible to answer in our study, but important to investigate in coming research, is: are there many patients “out there” who are never let through to an assessment for DBS?

Being well informed about a disease and its treatment might be empowering for patients, irrespective of diagnosis [24]. Hardly surprising, our patients’ own insights about PD and DBS were crucial and their considerations were related to their understanding. Knowledgeable patients had better abilities to ask or argue for DBS, and their insights reasonably increased their chances to be referred to a DBS team. Newspapers, TV and the Internet had been important, but the most significant information source was the PD-society, its meetings and journal. Reflecting on our results, we suggest that the best guarantee for PD-patients to keep updated about treatment options and new knowledge is to join a PD-society.

Similar to most countries, the acknowledged model for patient-doctor interaction in the Swedish healthcare system is patient-centred and emphasizes good communication, caring and trust. A goal is also that patients take part in choosing and deciding on alternative treatments [25,26]. However, even if shared decision-making is acknowledged as important, research shows that many physicians do not practice this approach on a regular basis [27,28]. On the other hand, research also shows that when patients ask questions or initiate discussions about treatment options, physicians respond with greater patient involvement in the decision process [29]. Maybe that was what happened for the patients in the subcategory ‘simply asking’: their questions were welcome by the physicians who explained more, and did send a referral to a DBS team. Not all of these patients decided to go ahead with operation at once, but being well informed and having had the chance to see a neurosurgeon for discussion was empowering. They felt included in “a helping plan” which increased their trust in healthcare and in clinicians, and reduced their despair and worries [30].

If the patient-doctor interaction had been smooth and focused on the best care for each patient, we believe that ‘Agreeing when offered’ should have been the main approach to DBS. Fewer patients would have had to initiate the discussion about DBS themselves, because their clinician would have done it, and none with severe symptoms despite adequate medication should have had to argue and struggle in order to be referred to a DBS team. That some patients ‘hesitate and wait’ seems to be reasonable within a discourse of shared decision-making; not all patients agree to their doctor’s suggestions, and the fact that some patients decline is a clear sign of their influence in the decision-making.

### Gender similarities and differences

Men and women were represented in all three approaches, and they considered similar issues on their path towards the decision to undergo neurosurgery. Two gender specific dimensions were identified in the patients’ considerations: *own technical skills* and *the exclusiveness of DBS* facilitated the decision process for a few of the men, but did not appear in the women’s narratives.

It has been previously suggested that the uneven gender distribution in neurosurgery for PD might be due to behavioural differences between men and women [6,14,17]. Such interpretations are in line with common notions about women being help-seeking and submissive [31], and, compared to male patients, it is uncommon that women demand and make a case for certain treatments. Looking at the experiences of the four women “taking the initiative” in our study (Ms Seventeen - Ms Twenty), we notice that Ms Twenty was more or less directly referred for assessment when she asked her clinician about DBS. The other three women were denied referral despite ‘demanding and arguing’. They succeeded to see a DBS team when they were supported by a PD-expert visiting the PD-society (Ms Seventeen); a friend who contacted another hospital (Ms Eighteen); or a daughter who forced the doctor to send a referral (Ms Nineteen). The men ‘demanding and arguing’ did it more on their own – at least they described it in such a way–. So, indeed, our findings support previous research [6,14], in that the gender gap in DBS might be related to men behaving more autonomously and more demanding than women, who rely instead more on support from other people. An alternative interpretation focuses on the doctors’ attitude and potential gender bias and suggests that the clinicians were more reluctant to listen to the women’s claims and needs. To obtain a referral to a DBS team, support from other people might therefore have been necessary for some women. Such an interpretation is in line with the fact that among the patients ‘taking own initiative’ for DBS, 44% (7/16) of the men belonged to the subcategory ‘simply asking’ and were met with a positive attitude from the clinician, compared to 25% (1/4) of the women who initiated DBS.

In line with previous suggestions [6,14,17], the gender distribution among the patients ‘hesitating and waiting’ indicates that strong fear for surgical risks is more common among women and could contribute to the gender disparities in DBS. However, this finding also creates new questions: are there ways to improve support for patients who are very apprehensive towards neurosurgery and DBS? To reason in line with Katz (2001), the fact that patients’ preferences and behaviour may contribute to gender disparities should not be construed as a signal that everything is as it should be [32]. Instead, clinicians should determine whether patients’ assumptions about risks and benefits of interventions are accurate or not, and misunderstandings should be corrected.

### The impact of other social determinants than gender

A recent European review concluded that there are still persistent and widespread inequities in health, both between and within countries, and that these arise from inequities in the distribution of power, money, and resources [33]. These social determinants of health intersect with gender and, therefore, can affect men and women differently [34]. In Sweden and elsewhere, fundamental social differences exist in the way that women and men are valued and treated and in the resources and resilience they possess [35,36].

In light of this, it is not surprising that in addition to gender, other social determinants seemed to be entangled in the gendered pattern among our participants. Our results suggest that both societal position and formal education were related to the patient’s approach to DBS. In patients ‘taking own initiative’ for DBS, the level of education was higher than in the other two categories and about 80% described having been in some kind of leadership position in the society, compared to 33% among those ‘agreeing when offered’, and none among the ‘hesitating’ patients. A patient with education or social capital from leadership structures implies a more “powerful” patient, with more expectation and demands for being listened to. If not met with attention, a patient with high self-reliance has more resources to pursue his or her case. To strive for equity in healthcare and to counter social power gradients, physicians need to be especially aware of the needs among the powerless, the less educated and among social groups like women who have traditionally taken a submissive position in society.

### On method

Qualitative studies do not claim for generalizability. Instead the term transferability is used. Transferability to other contexts has to be done with some carefulness [21,37]. Differences in gender equality, in norms and habits in the patient-doctor relationship, the organisation of healthcare, the economic costs for the individual, as well as disparities in the opportunities to turn to private care for DBS, are some examples of circumstances that might compromise the transferability of our findings. This study was conducted in northern Sweden but we have no reason to believe that patients from the rest of Scandinavia would approach DBS in other ways. Transferability to other countries has to be done with more caution. With the information given about our setting and participants, it is up to the readers to assess whether our findings can be useful in their situation.

A strength of this study was that we invited all patients treated with DBS at our university hospital during a certain time span. Since one aim was to explore the impact of gender on patients’ considerations about surgery, the low number of women available for interview was a weakness. In an attempt to achieve a more gender-balanced sample, we therefore searched for additional women operated elsewhere and were able to include three more women. Before doing this, we weighted the need for more data against possible bias that might infiltrate since the three new women came from other parts of Sweden and their recruitment was done with help of the Parkinson Disease society. It might be, for example, that these women were very special – perhaps very knowledgeable, active and pushing. However, their narratives and experiences fitted well into the established categories. The additional interviews contributed with valuable examples congenerous with the experiences of the patients operated in Umeå, which strengthened the trustworthiness of the analysis [21,37].

The concept of a ‘leadership position’ used in this study is a compound concept that is grounded in the data and outlined during the analysis. It cannot be reduced to, for example, having high education or holding managerial positions at work. We realized that some participants with low formal education, or modest work titles, still had good communication skills and knowledge about how to argue and convey their point. In the case of these patients, these competencies were related to previous commitments in trade unions or non-governmental organisations. We suggest that this compound concept of ‘leadership position’ should be further explored and developed in a quantitative setting.

All participants in our study were operated and treated with DBS and our findings cannot be straight away transferred to patients with advanced PD who have not undergone surgery. Even if we believe that ‘taking own initiative’, ‘agreeing when offered’ and ‘hesitating and waiting’ are valid approaches, interviewing non-operated patients might reveal also other experiences and additional approaches that do not end up in a decision to undergo neurosurgery. We also do not know to what extent patients with advanced PD are persistently denied DBS assessment when they ask for it, or how common it is that patients who are eligible for DBS according to medical criteria, but who themselves do not argue for neurosurgery, are neglected and never suggested for operation, i.e. are never offered the chance to ‘agree’. To investigate these issues, further research is needed about experiences among patients with advanced PD who have not undergone surgery.

## Conclusion

This study suggests that it is common that patients with advanced PD have to argue and struggle to convince their clinicians to refer them for an assessment by a DBS team. This creates uncertainty among patients and may lead to unequal care, where patients with privileged socio-economic positions and high self-reliance are favoured. The results further illustrate that patients’ own wishes as well as position in society may contribute to a skewed gender distribution among patients who receive DBS for PD. More women than men described worries about complications and this fear kept some women from undergoing surgery for several years. Some men were very tempted by the exclusiveness and advanced technology used in DBS. Being able to communicate effectively and to demand and argue one’s position was important for the patients to get referral for an assessment for DBS. Such competencies are developed and strengthened among leaders of any organisation, and the fact that overall more men than women hold leading positions in the society implies that ‘leadership position’ intersects with ‘gender’ and contributes to gender differences.

To strive for equity in healthcare and counter social power gradients, physicians need to be especially aware of the needs among the powerless, the less educated, and among social groups, such as women, that tend to take a submissive position in society.

## Competing interest

The authors declare that they have no competing interests.

## Authors’ contributions

KH and G-MH equally contributed to the conception and design, data-collection and analysis. KH took the main responsibility for drafting the manuscript. Both authors contributed substantially with critical comments and intellectual content throughout the writing process and both have given approval of the submitted manuscript.

## Pre-publication history

The pre-publication history for this paper can be accessed here:

http://www.biomedcentral.com/1471-2377/14/89/prepub
